# The Value of Emergency Care Data Set (ECDS) Presentation Codes for Predicting Mortality and Inpatient Admission

**DOI:** 10.7759/cureus.56083

**Published:** 2024-03-13

**Authors:** Betsy Teresa, Mohammed Subhi, Adrian Boyle, Wayne Kark

**Affiliations:** 1 Emergency Medicine, Cambridge University Hospitals National Health Service (NHS) Foundation Trust, Cambridge, GBR; 2 General Practice, Staploe Medical Centre, Cambridge, GBR

**Keywords:** ecds, triage, presenting complaints, news2 score, hospital admission, mortality

## Abstract

Background: Early identification of patients at higher risk of death and hospital admission is an important problem in Emergency Departments (ED). Most triage scales were developed before current electronic healthcare records were developed. The implementation of a national Emergency Care Data Set (ECDS) allows for the standardised recording of presenting complaints and the use of Electronic Patient Records (EPR) offers the potential for automated triage. The mortality risk and need for hospital admission associated with the different presenting complaints in a standardised national data set has not been previously reported. This study aimed to quantify the risks of death and hospitalisation from presenting complaints. This would be valuable in developing automated triage tools and decision support software.

Methods: We conducted an observational retrospective cohort study on patients who visited a single ED in 2021. The presenting complaints related to subsequent attendances were excluded. This patient list was then manually matched with a routinely collected list of deaths. All deaths that occurred within 30 days of attendance were included.

Results: Data was collected from 84,999 patients, of which 1,159 people died within 30 days of attendance. The mortality rate was the highest in cardiac arrest [32 (78.1%)], cardiac arrest due to trauma [2(50%)] and respiratory arrest [3(50%)]. Drowsy [17(12%)], hypothermia [3(13%)] and cyanosis [1(10%)] were also high-risk categories. Chest pain [34(0.6%)] was not a high-risk presenting complaint.

Conclusion: The initial presenting complaint in ECDS may be useful to identify people at higher and lower risk of death. This information is useful for building automated triage models.

## Introduction

Background

The Emergency Care Data Set (ECDS) was implemented in October 2017 and is the National Health Service (NHS) standard for Emergency Care in England [1]. The ECDS is the national data set for urgent and emergency care which provides information on activity in Emergency Departments (ED). All EDs in England mandatorily use this standardised coding system to record data on all its attendances which is critical for clinical/operational reporting. The presenting complaint of the patient is standardised within ECDS [2]. Early identification of serious illness and injury is a key function of triage at initial assessment. Currently, there are several triage scales in use, none of which have demonstrated superiority, or non-inferiority. Most triage scales were developed for paper notes [3]. Electronic health records raise the possibility of automated identification of patients at risk of serious harm.

Acuity may be determined by a triage process augmented with an early warning score (NEWS2) or by the physical allocation of the patient to a specific clinical area such as resuscitation [4]. National Early Warning Score (NEWS2) is the second iteration of the NEWS score which is a tool for scoring physiological measurements already recorded in routine practice. Studies have shown that NEWS2 is valid for predicting death for patients within the ED setting [5]. With increasingly crowded EDs, it is important to be able to rapidly and efficiently identify people at the greatest risk of death [6,7]. Operationally, it is also useful to identify which patients are at risk of hospital admission early, as this allows earlier bed requests.

NEWS2 scores are based on vital signs in adults and represent a patient’s clinical state at that time and provide some useful prediction about the risk of death and need for hospitalisation, but are not perfect [8]. For instance, the NEWS2 in patients with cardiovascular diseases is suboptimal to predict deterioration early [9].

The mortality risks and need for hospital admission associated with the initial presenting complaint in a standardised national data set have not been previously reported. Chest pain might sound dangerous to an external observer due to concerns about the possibility of myocardial infarction, though many emergency physicians will recognise that there are a lot of people with chest pain whose risk of death is very low [10]. Likewise, generalised weakness is a nonspecific symptom that may be encountered in a large number of medical and psychiatric disorders [11] which anecdotally may be associated with a higher risk of death and hospital admission. Quantifying the risk of death and hospital admission would be useful for developing automated triage tools and decision-support software.

Objectives

We aimed to quantify the mortality and risk of hospital admission associated with the presenting complaint in a standardised national coding system. As a secondary objective, we aimed to investigate the value of the initial NEWS2 score recorded at the time of ED presentation in predicting all-cause mortality within 30 days of ED admission. The overall aim was to evaluate the utility of ECDS reason for presentation as a potential triage and workload prioritisation tool.

We also evaluated the risks associated with the Royal College of Emergency Medicine (RCEM) standards for consultant sign-off; these are presentations where a consultant should review a patient before discharge. RCEM is an independent professional association of emergency physicians in the United Kingdom. Consultants are senior doctors who have completed full medical training in Emergency Medicine and are listed on the General Medical Council's specialist register.

This data would be useful for building machine learning and artificial intelligence models for decision-support tools, specifically an automated triage tool. Practically, identifying a cohort of people at very low risk of death could be useful for safe redirection to other services or self-care.

## Materials and methods

Study design and setting

We conducted an observational retrospective cohort study on patients who visited the ED at Addenbrooke’s Hospital, Cambridge, UK from 1st January 2021 to 31st of December 2021. Addenbrooke’s Hospital provides emergency, surgical and medical care for local people and is the major trauma centre (MTC) for the East of England. We took a 12-month sample so as to mitigate seasonal fluctuations in mortality and presentations.

Participants

All patients, including children, seeking ED care at Addenbrooke’s Hospital were included in this study. Data regarding the initial presentation of the patient in 2021 was included in the study. We excluded presenting complaints related to subsequent attendances, as this avoids the problem of multiple exposures. This patient list was matched with a routinely collected list of deaths occurring after attendance at the ED. There were no further exclusion criteria. The list of people who died is part of the Patient Administration System (PAS) and receives notifications from the inpatient team if they die in hospital and from the patient’s General Practitioner if they die out of hospital. Finally, each week an administrator checks any patients who are receiving ‘active care’ such as future planned care or recent attendance, from the hospital against the NHS Spine, a centralised register of deaths.

Data sources and variables

Data was obtained from the Trust’s Electronic Patient Record (EPR), which is used to record all patient contacts during their stay in the ED. All of this information is subsequently stored in a centralised database. The list of attendances was manually matched to the list of deaths. We included all deaths that occurred within 30 days of attendance, regardless of admission or discharge from the ED. We also recorded the ECDS reason for attendance, this is routinely recorded by nursing staff at triage. There is no facility at our institution for patients to self-register or record their own reason for presentation. We also report routinely collected data on potential confounding variables including age, sex, arrival by ambulance and NEWS2 score (in adults only). We dichotomised the disposition code into admission or discharge.

## Results

Participants data

A total of 121,099 patients presented to the ED during the study period, data were collected from 84,999 patients after excluding all the repeated attendances to the ED. About 1159 (1.36%) people died within 30 days of attendance at the ED. The characteristics of the patients are shown in Table 1.

**Table 1:** Descriptive summary of sample data Data are shown as n or %.

Age (Years)	No. (n)	Percentage (%)
0-4	7974	9.38
5-14	7775	9.15
15-24	11351	13.35
25-34	11345	13.35
35-44	9265	10.9
45-54	8900	10.47
55-64	8280	9.74
65-74	7658	9.01
75-84	7356	8.65
85-94	4485	5.28
95-104	586	0.69
>104	24	0.03
Total	84999	100
Gender		
Male	42030	49.45
Female	42932	50.51
Non-binary	15	0.02
Unknown	22	0.03
Total	84999	100
Arrival mode		
Ambulance	11491	13.52
Other methods	73508	86.48
Total	84999	100

Frequency and outcomes of presenting complaints

The most frequent presenting complaints were injury of shoulder/arm/elbow/wrist/hand in 8416 (9.9%), injury of hip/leg/knee/ankle/foot in 7427 (8.7%), chest pain in 5953 (7%) and abdominal pain in 5629 (6.6%) of 84999 patients. The percentage of hospital admission (100%) and mortality were the highest in the following categories: Cardiac arrest [22 (53.7%); 32 (78.1%)], cardiac arrest due to trauma [1 (25%); 2 (50%)] and respiratory arrest [2 (33.3%); 3 (50%)] consistently within day 1 and day 30 of the first presentation. Although chest pain and abdominal pain are among the most common presentations, they contributed less than 1% to the mortality rate.

Drowsy [17 (12%)], hypothermia [3 (13%)] and cyanosis [1 (10%)] are the categories that followed closely behind with a high 30-day in-hospital mortality rate. Tables 2-4 showed that the mortality rates differed per presenting complaint.

**Table 2:** Proportion of deaths by one and 30 days and admission in patients categorised by the presenting complaints - groups at high risk Data are shown as n, % [CI] or %. CI = Confidence Interval

Presenting Complaint	Total First Presentations Over 2021 (%)	Deaths by Day 1 (n)	% [95% CI]	Deaths by Day 30 (n)	% [95% CI]	Admission (n)	Percentage (%)
Total:	84999 (100)	160	0.2[0.2, 0.2]	1159	1.4[1.3, 1.4]	25395	29.9
Short of Breath	3735 (4.4)	29	0.8[0.5, 1.1]	227	6.1[5.4, 6.9]	2446	65.5
Cardiac Arrest	41 (0)	22	53.7[38.8, 67.9]	32	78.1[63.3, 88.0]	41	100
Major Trauma (Serious Injury >1 Body Area)	474 (0.6)	15	3.2[1.9, 5.2]	34	7.2[5.2, 9.9]	406	85.7
Collapse/Fainting Episode	1098 (1.3)	12	1.1[0.6, 1.9]	38	3.5[2.5, 4.7]	498	45.4
Generalised Weakness	1831 (2.2)	8	0.4[0.2, 0.9]	119	6.5[5.5, 7.7]	1085	59.3
Collapse/Fainting Episode With Loss of Consciousness	644 (0.8)	8	1.2[0.6, 2.4]	37	5.8[4.2, 7.8]	302	46.9
Head Injury	3493 (4.1)	7	0.2[0.1, 0.4]	22	0.6[0.4, 1.0]	382	10.9
Chest Pain	5953 (7)	5	0.1[0.0, 0.2]	34	0.6[0.4, 0.8]	2077	34.9
Abdominal Pain	5629 (6.6)	5	0.1[0.0, 0.2]	48	0.9[0.6, 1.1]	2473	43.9
Seizure (Fit)	715 (0.8)	5	0.7[0.3, 1.6]	16	2.2[1.4, 3.6]	250	35
Facial Weakness	368 (0.4)	5	1.4[0.6, 3.1]	25	6.8[4.6, 9.8]	211	57.3
Fever	2931 (3.4)	4	0.1[0.1, 0.4]	53	1.8[1.4, 2.4]	1065	36.3
Falls/Unsteady On Feet	2231 (2.6)	3	0.1[0.1, 0.4]	111	5[4.1, 6.0]	1294	58
Confusion	415 (0.5)	3	0.7[0.3, 2.1]	29	7[4.9, 9.9]	325	78.3
Unknown	270 (0.3)	3	1.1[0.4, 3.2]	9	3.3[1.8, 6.2]	73	27
Drowsy (Altered Level of Consciousness)	142 (0.2)	3	2.1[0.7, 6.0]	17	12[7.6, 18.3]	96	67.6
Direct Referral to Inpatient Unit	2047 (2.4)	2	0.1[0.0, 0.4]	65	3.2[2.5, 4.0]	1391	68
Difficulty Breathing	1032 (1.2)	2	0.2[0.1, 0.7]	15	1.5[0.9, 2.4]	311	30.1
Limb Weakness	481 (0.6)	2	0.4[0.1, 1.5]	32	6.7[4.8, 9.2]	325	67.6
Speech Disturbance	378 (0.4)	2	0.5[0.2, 1.9]	12	3.2[1.8, 5.5]	276	73
Respiratory Arrest	6 (0)	2	33.3[9.7, 70.0]	3	50[18.8, 81.2]	6	100
Backache (No Recent Injury)	1548 (1.8)	1	0.1[0.0, 0.4]	7	0.5[0.2, 0.9]	331	21.4
Palpitations	1473 (1.7)	1	0.1[0.0, 0.4]	8	0.5[0.3, 1.1]	554	37.6
Vomiting +/- Nausea	1112 (1.3)	1	0.1[0.0, 0.5]	11	1[0.6, 1.8]	439	39.5
Dizziness	897 (1.1)	1	0.1[0.0, 0.6]	4	0.5[0.2, 1.1]	309	34.5
Throat: Cough	896 (1.1)	1	0.1[0.0, 0.6]	6	0.7[0.3, 1.5]	232	25.9
Vaginal Bleeding (Abnormal)	559 (0.7)	1	0.2[0.0, 1.0]	1	0.2[0.0, 1.0]	178	31.8
Blood In Stools	521 (0.6)	1	0.2[0.0, 1.1]	7	1.3[0.7, 2.7]	247	47.4
Diarrhoea	435 (0.5)	1	0.2[0.0, 1.3]	9	2.1[1.1, 3.9]	211	48.5
Pain on Passing Urine	194 (0.2)	1	0.5[0.1, 2.9]	2	1[0.3, 3.7]	45	23.2
Vomiting Blood	186 (0.2)	1	0.5[0.1, 3.0]	9	4.8[2.6, 8.9]	96	51.6
Hypoglycaemia	76 (0.1)	1	1.3[0.2, 7.1]	3	4[1.4, 11.0]	44	57.9
Hypothermia	23 (0)	1	4.4[0.8, 21.0]	3	13[4.5, 32.1]	11	47.8
Cardiac Arrest Due to Trauma	4 (0)	1	25[4.6, 69.9]	2	50[15.0, 85.0]	4	100
Injury of Shoulder/Arm/Elbow/Wrist/Hand	8416 (9.9)	0	0[0.0, 0.1]	4	0.1[0.0, 0.1]	424	5
Injury of Hip/Leg/Knee/Ankle/Foot	7427 (8.7)	0	0[0.0, 0.1]	15	0.2[0.1, 0.3]	653	8.8
Pain in Hip/Leg/Knee/Ankle/Foot	2079 (2.4)	0	0[0.0, 0.2]	7	0.3[0.2, 0.7]	396	19.1
Headache	1859 (2.2)	0	0[0.0, 0.2]	6	0.3[0.2, 0.7]	583	31.4
Localised Swelling/Redness/Lumps/Bumps	1594 (1.9)	0	0[0.0, 0.2]	10	0.6[0.3, 1.2]	450	28.2
Facial Injury	1555 (1.8)	0	0[0.0, 0.3]	1	0.1[0.0, 0.4]	128	8.2
Rash	935 (1.1)	0	0[0.0, 0.4]	1	0.1[0.0, 0.6]	109	11.7
Self-Harm	906 (1.1)	0	0[0.0, 0.4]	2	0.2[0.1, 0.8]	309	34.1
Wound: Laceration	894 (1.1)	0	0[0.0, 0.4]	0	0[0.0, 0.4]	131	14.7
Pain in Shoulder/Arm/Elbow/Wrist/Hand	805 (0.9)	0	0[0.0, 0.5]	1	0.1[0.0, 0.7]	106	13.2
Flank Pain	774 (0.9)	0	0[0.0, 0.5]	1	0.1[0.0, 0.7]	211	27.3
Visual Disturbance	757 (0.9)	0	0[0.0, 0.5]	0	0[0.0, 0.5]	327	43.2
Pain in/Around Eye	640 (0.8)	0	0[0.0, 0.6]	0	0[0.0, 0.6]	155	24.2
Throat: Sore	598 (0.7)	0	0[0.0, 0.6]	2	0.3[0.1, 1.2]	157	26.3

**Table 3:** Proportion of deaths by one and 30 days and admission in patients categorised by the presenting complaints - groups at intermediate risk Data are shown as n, % [CI] or %. CI = Confidence interval

Presenting Complaint	Total First Presentations Over 2021 (%)	Deaths by Day 1 (n)	% [95% CI]	Deaths by Day 30 (n)	% [95% CI]	Admission (n)	Percentage (%)
Total:	84999 (100)	160	0.2[0.2, 0.2]	1159	1.4[1.3, 1.4]	25395	29.9
Foreign Body in Eye	589 (0.7)	0	0[0.0, 0.7]	0	0[0.0, 0.7]	48	8.2
Facial Pain (Inc. Toothache)	574 (0.7)	0	0[0.0, 0.7]	0	0[0.0, 0.7]	107	18.6
Injury of Lower Back	573 (0.7)	0	0[0.0, 0.7]	1	0.2[0.0, 1.0]	76	13.3
Postoperative/Medical Device With Complication	571 (0.7)	0	0[0.0, 0.7]	4	0.7[0.3, 1.8]	221	38.7
Eye Injury	498 (0.6)	0	0[0.0, 0.8]	0	0[0.0, 0.8]	81	16.3
Burn	478 (0.6)	0	0[0.0, 0.8]	0	0[0.0, 0.8]	35	7.3
Ear: Pain	449 (0.5)	0	0[0.0, 0.9]	0	0[0.0, 0.9]	65	14.5
Drug/Alcohol Intoxication or Withdrawal	438 (0.5)	0	0[0.0, 0.9]	0	0[0.0, 0.9]	60	13.7
Wound: Bite	419 (0.5)	0	0[0.0, 0.9]	0	0[0.0, 0.9]	49	11.7
Swollen Leg (Single)	414 (0.5)	0	0[0.0, 0.9]	2	0.5[0.1, 1.7]	156	37.7
Unable to Pass Urine	398 (0.5)	0	0[0.0, 1.0]	6	1.5[0.7, 3.2]	130	32.7
Neck Pain	376 (0.4)	0	0[0.0, 1.0]	1	0.3[0.0, 1.5]	40	10.6
Numbness/Tingling (Paresthesia)	348 (0.4)	0	0[0.0, 1.1]	0	0[0.0, 1.1]	71	20.4
Nose: Bleeding From Nose	342 (0.4)	0	0[0.0, 1.1]	1	0.3[0.1, 1.6]	74	21.6
Pain In Scrotum/Testes	311 (0.4)	0	0[0.0, 1.2]	1	0.3[0.1, 1.8]	109	35.1
Eye Review	301 (0.4)	0	0[0.0, 1.3]	0	0[0.0, 1.3]	96	31.9
Suicidal Thoughts	282 (0.3)	0	0[0.0, 1.3]	0	0[0.0, 1.3]	60	21.3
Injury of Thorax	266 (0.3)	0	0[0.0, 1.4]	0	0[0.0, 1.4]	37	13.9
Pain in Genital Area (Generalised)	262 (0.3)	0	0[0.0, 1.5]	0	0[0.0, 1.5]	69	26.3
Blood in Urine	247 (0.3)	0	0[0.0, 1.5]	1	0.4[0.1, 2.3]	84	34
Ear: Foreign Body	225 (0.3)	0	0[0.0, 1.7]	0	0[0.0, 1.7]	19	8.4
Wound: Sting	200 (0.2)	0	0[0.0, 1.9]	0	0[0.0, 1.9]	16	8
Poisoning From Any Source	179 (0.2)	0	0[0.0, 2.1]	0	0[0.0, 2.1]	8	4.5
Jaundice	178 (0.2)	0	0[0.0, 2.1]	7	3.9[1.9, 7.9]	105	59
Constipation	171 (0.2)	0	0[0.0, 2.2]	1	0.6[0.1, 3.2]	52	30.4
Postoperative/Wound Care (No Complication)	168 (0.2)	0	0[0.0, 2.2]	0	0[0.0, 2.2]	49	29.2
Foreign Body in Digestive Tract	164 (0.2)	0	0[0.0, 2.3]	0	0[0.0, 2.3]	19	11.6
Hyperglycaemia	162 (0.2)	0	0[0.0, 2.3]	6	3.7[1.7, 7.8]	119	73.5
Coughing Up Blood (Haemoptysis)	156 (0.2)	0	0[0.0, 2.4]	1	0.6[0.1, 3.5]	70	44.9
Red Eye	145 (0.2)	0	0[0.0, 2.6]	0	0[0.0, 2.6]	33	22.8
Noisy Breathing	144 (0.2)	0	0[0.0, 2.6]	0	0[0.0, 2.6]	17	11.8
Behaviour: Unusual	142 (0.2)	0	0[0.0, 2.6]	1	0.7[0.1, 3.9]	52	36.6
Injury of Upper Back	136 (0.2)	0	0[0.0, 2.8]	0	0[0.0, 2.8]	12	8.8
Abdominal Distension	131 (0.2)	0	0[0.0, 2.9]	5	3.8[1.6, 8.6]	79	60.3
Foreign Body in Skin/Subcutaneous Tissue	131 (0.2)	0	0[0.0, 2.9]	0	0[0.0, 2.9]	12	9.2
Injury of Neck	128 (0.2)	0	0[0.0, 2.9]	0	0[0.0, 2.9]	13	10.2
Loss of Appetite	128 (0.2)	0	0[0.0, 2.9]	0	0[0.0, 2.9]	55	43
Anxiety Disorder	125 (0.1)	0	0[0.0, 3.0]	0	0[0.0, 3.0]	17	13.6
Swollen Legs (Both)	120 (0.1)	0	0[0.0, 3.1]	5	4.2[1.8, 9.4]	76	63.3
Pregnancy-Related: Less Than 20 Weeks	120 (0.1)	0	0[0.0, 3.1]	0	0[0.0, 3.1]	42	35
Rectal Pain	114 (0.1)	0	0[0.0, 3.3]	0	0[0.0, 3.3]	40	35.1
Frequent Urination	113 (0.1)	0	0[0.0, 3.3]	3	2.7[0.9, 7.5]	49	43.4
Exposure to Communicable Disease (Inc. Needlestick/Body Fluids)	113 (0.1)	0	0[0.0, 3.3]	0	0[0.0, 3.3]	12	10.6
Crying Infant	108 (0.1)	0	0[0.0, 3.4]	0	0[0.0, 3.4]	16	14.8
Throat: Foreign Body in Throat/Mouth	108 (0.1)	0	0[0.0, 3.4]	0	0[0.0, 3.4]	18	16.7
Joint Swelling	103 (0.1)	0	0[0.0, 3.6]	0	0[0.0, 3.6]	28	27.2
Nose: Foreign Body	100 (0.1)	0	0[0.0, 3.7]	0	0[0.0, 3.7]	4	4
Nose: Injury	100 (0.1)	0	0[0.0, 3.7]	0	0[0.0, 3.7]	2	2

**Table 4:** Proportion of deaths by one and 30 days and admission in patients categorised by the presenting complaints - groups at low risk Data are shown as n, % [CI] or %. CI = Confidence interval

Presenting Complaint	Total First Presentations Over 2021 (%)	Deaths by Day 1 (n)	% [95% CI]	Deaths by Day 30 (n)	% [95% CI]	Admission (n)	Percentage (%)
Total:	84999 (100)	160	0.2[0.2, 0.2]	1159	1.4[1.3, 1.4]	25395	29.9
Problem Related to Penis	99 (0.1)	0	0[0.0, 3.7]	1	1[0.2, 5.5]	8	8.1
Cold Painful Limb	88 (0.1)	0	0[0.0, 4.2]	4	4.6[1.8, 11.1]	57	64.8
Behaviour: Agitated/Violent	87 (0.1)	0	0[0.0, 4.2]	1	1.2[0.2, 6.2]	42	48.3
Requesting Prescription	84 (0.1)	0	0[0.0, 4.4]	0	0[0.0, 4.4]	17	20.2
Food/Foreign Body in Oesophagus	80 (0.1)	0	0[0.0, 4.6]	0	0[0.0, 4.6]	24	30
Wound: Puncture	78 (0.1)	0	0[0.0, 4.7]	1	1.3[0.2, 6.9]	26	33.3
Low Urine Output	77 (0.1)	0	0[0.0, 4.8]	2	2.6[0.7, 9.0]	33	42.9
Itching	77 (0.1)	0	0[0.0, 4.8]	0	0[0.0, 4.8]	3	3.9
Abnormal Swelling Groin Area	76 (0.1)	0	0[0.0, 4.8]	0	0[0.0, 4.8]	31	40.8
Injury of Abdomen	70 (0.1)	0	0[0.0, 5.2]	0	0[0.0, 5.2]	19	27.1
Ear: Hearing Loss	65 (0.1)	0	0[0.0, 5.6]	0	0[0.0, 5.6]	10	15.4
Ear: Injury	65 (0.1)	0	0[0.0, 5.6]	0	0[0.0, 5.6]	6	9.2
Noxious Inhalation - Gas/Fumes/Vapour/Smoke	63 (0.1)	0	0[0.0, 5.8]	0	0[0.0, 5.8]	1	1.6
Problem-Related to Vagina	61 (0.1)	0	0[0.0, 5.9]	0	0[0.0, 5.9]	19	31.2
Discharge From Eye	59 (0.1)	0	0[0.0, 6.1]	0	0[0.0, 6.1]	9	15.3
Problem Related To Breast	59 (0.1)	0	0[0.0, 6.1]	0	0[0.0, 6.1]	15	25.4
Social Problem (Medically Well)	58 (0.1)	0	0[0.0, 6.2]	1	1.7[0.3, 9.1]	27	46.6
Nausea Without Vomiting	55 (0.1)	0	0[0.0, 6.5]	0	0[0.0, 6.5]	14	25.5
Chemical Exposure	48 (0.1)	0	0[0.0, 7.4]	0	0[0.0, 7.4]	1	2.1
Wound: Abrasion	46 (0.1)	0	0[0.0, 7.7]	0	0[0.0, 7.7]	13	28.3
Ear: Discharge	45 (0.1)	0	0[0.0, 7.9]	0	0[0.0, 7.9]	6	13.3
Hallucinations/Delusions	44 (0.1)	0	0[0.0, 8.0]	1	2.3[0.4, 11.8]	19	43.2
Foreign Body in Respiratory Tract	43 (0.1)	0	0[0.0, 8.2]	1	2.3[0.4, 12.1]	9	20.9
Depressive Disorder	36 (0)	0	0[0.0, 9.6]	0	0[0.0, 9.6]	8	22.2
Foreign Body in Vagina	34 (0)	0	0[0.0, 10.2]	0	0[0.0, 10.2]	10	29.4
Tremor	33 (0)	0	0[0.0, 10.4]	0	0[0.0, 10.4]	15	45.5
Injury to Genital Area	32 (0)	0	0[0.0, 10.7]	0	0[0.0, 10.7]	4	12.5
Pale Colour	27 (0)	0	0[0.0, 12.5]	0	0[0.0, 12.5]	18	66.7
Spontaneous Bruising	22 (0)	0	0[0.0, 14.9]	0	0[0.0, 14.9]	5	22.7
Injury of Cervical Region of Back (Disorder)	21 (0)	0	0[0.0, 15.5]	0	0[0.0, 15.5]	6	28.6
Nose: Congestion	20 (0)	0	0[0.0, 16.1]	0	0[0.0, 16.1]	3	15
Ear: Ringing in Ears (Tinnitus)	17 (0)	0	0[0.0, 18.4]	0	0[0.0, 18.4]	3	17.7
Electrical Exposure (Inc. Lightning)	14 (0)	0	0[0.0, 21.5]	0	0[0.0, 21.5]	1	7.1
Pregnancy-Related: Greater Than 20 Weeks	14 (0)	0	0[0.0, 21.5]	0	0[0.0, 21.5]	10	71.4
Foreign Body in Rectum	13 (0)	0	0[0.0, 22.8]	0	0[0.0, 22.8]	4	30.8
Blue Colour (Cyanosis)	10 (0)	0	0[0.0, 27.8]	1	10[1.8, 40.4]	4	40
Photophobia	9 (0)	0	0[0.0, 29.9]	0	0[0.0, 29.9]	4	44.4
Infant With Episodes Not Breathing (Apnoea)	8 (0)	0	0[0.0, 32.4]	0	0[0.0, 32.4]	3	37.5
States Victim of Sexual Assault	8 (0)	0	0[0.0, 32.4]	0	0[0.0, 32.4]	0	0
Traumatic Amputation	7 (0)	0	0[0.0, 35.4]	0	0[0.0, 35.4]	4	57.1
Injury of Anus	6 (0)	0	0[0.0, 39.0]	0	0[0.0, 39.0]	2	33.3
Insomnia	5 (0)	0	0[0.0, 43.5]	0	0[0.0, 43.5]	0	0
Near Drowning	5 (0)	0	0[0.0, 43.5]	0	0[0.0, 43.5]	2	40
Neurologic Problem	2 (0)	0	0[0.0, 65.8]	0	0[0.0, 65.8]	2	100
Consultant Review	1 (0)	0	0[0.0, 79.4]	0	0[0.0, 79.4]	1	100
Decreased Fetal Movement	1 (0)	0	0[0.0, 79.4]	0	0[0.0, 79.4]	1	100
Hiccoughs	1 (0)	0	0[0.0, 79.4]	0	0[0.0, 79.4]	0	0
Postnatal Assessment	1 (0)	0	0[0.0, 79.4]	0	0[0.0, 79.4]	1	100

**Figure 1:**
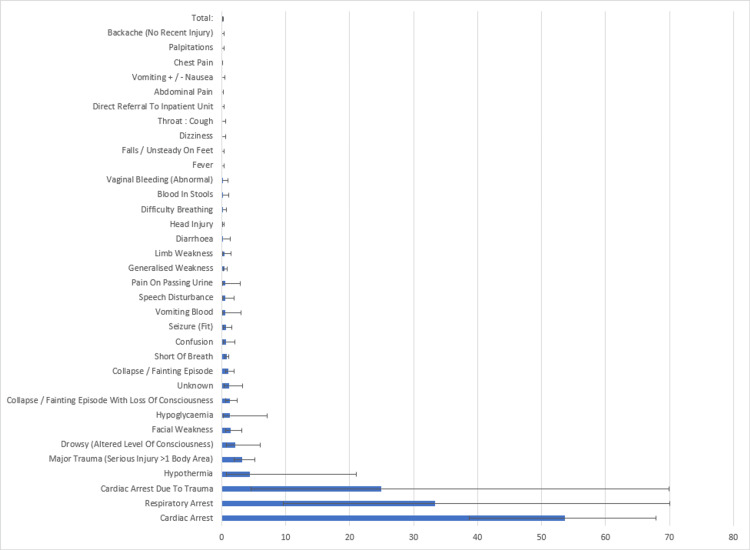
Probability of death within 24 hours by ECDS chief complaint (% [95% CI]) Data in the x-axis represents the deaths that occurred within 24 hours of attendance and are shown as % [CI]. The y-axis corresponds to the ECDS chief complaints. CI = Confidence interval, ECDS = Emergency Care Data Set

Figure 1 shows that there are three broad categories of risk related to mortality, high risk related to some form of cardiorespiratory arrest, intermediate where there is some (variable) mortality risk, and low risk where there is no mortality risk.

Analysis of selected patients who require senior review

Selected high-risk patient groups need to be reviewed by a consultant in Emergency Medicine prior to discharge according to standards published and updated by the RCEM in 2016 [12]. Analysis of this data depicted in Table 5 showed that the overall 30-day mortality rate was lower as compared to some of the other categories of presenting complaints recorded in Tables 2-4. The percentage of overall deaths was of note <1% (33) in patients aged 30 years or more presenting with chest pain and 0% (0) in febrile infants.

**Table 5:** Proportion of deaths by one and 30 days in patients who require consultant sign-off Data are shown as n or % [CI]. CI = Confidence Interval, RCEM = Royal College of Emergency Medicine

RCEM Signoff Criteria	Total First Presentations Over 2021	Deaths by Day 1 (n)	% [95% CI]	Deaths by Day 30 (n)	% [95% CI]
Abdo pain in >70y	870	5	0.6 [0.3,1.3]	37	4.3 [3.1, 5.8]
Chest pain in >30y	4926	5	0.1 [0.0,0.2]	33	0.7 [0.5,0.9]
Fever in <1y	900	0	0 [0.0,0.4]	0	0 [0.0,0.4]

Mode of arrival, NEWS2 score and mortality

**Figure 2:**
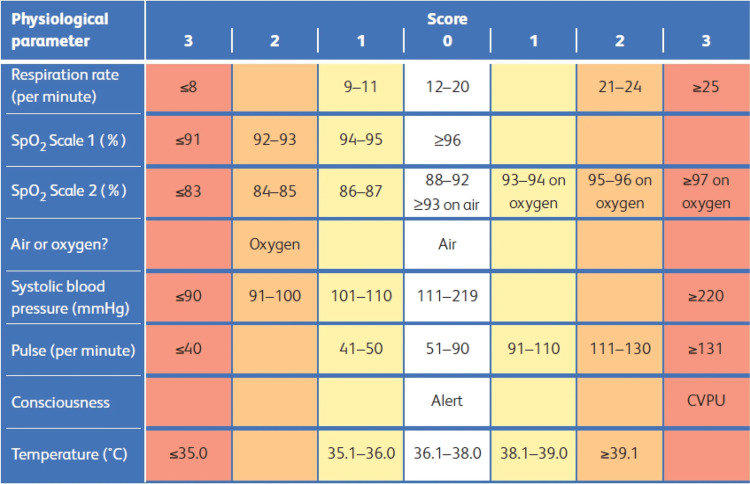
The NEWS2 scoring system NEWS = National Early Warning Score

NEWS2 is not recorded in people under the age of 16, and we included 68,444 adults in this analysis (Figure 2). Arrival mode was found to be strongly associated with an increased risk of mortality, with 48.9% (567) of the total deaths comprising patients who arrived by ambulance.

A tabulation of the proportions of patient deaths by one and 30 days revealed that the mortality rate steadily increased as the NEWS score increased (Table 6).

**Table 6:** Proportion of Deaths by one and 30 days in patients categorised by initial NEWS2 score and mode of arrival Data are shown as n or % [CI]. CI = Confidence interval, NEWS = National Early Warning Score

NEWS	Deaths by Day 1 (n)	% [95% CI]	Deaths by Day 30 (n)	% [95% CI]	Total Presentations
No score	52	0.1 [0.1,0.2]	71	0.2 (0.2,0.2)	36882
0	4	0 [0.0,0.1]	112	0.6 (0.5,0.7)	18618
1	9	0.1 [0.0,0.1]	149	1.1 (0.9,1.3)	13804
2	0	0 [0.0,0.1]	123	1.9 (1.6,2.2)	6609
3	4	0.1 [0.0,0.3]	111	3 (2.5,3.6)	3730
4	5	0.3 [0.1,0.6]	97	4.8 (4.0,5.8)	2015
5	8	0.7 [0.3,1.3]	88	7.5 (6.1,9.1)	1179
6	19	2.2 [1.4,3.4]	107	12.3 (10.3,14.7)	870
7	16	2.8 [1.7,4.4]	100	17.3 (14.4,20.6)	579
8	19	4.5 [2.9,6.8]	112	26.2 (22.3,30.6)	427
9	24	8.4 [5.7,12.2]	89	31.1 (26.0,36.7)	286
>9	0	N/A	0	N/A	0
Total	160		1159		84999
Mode of arrival					
Ambulance	82	0.7 [0.6,0.9]	567	4.9 (4.6,5.4)	11491
Other	78	0.1 [0.1,0.1]	592	0.8 (0.7,0.9)	73508
Total	160	0.2 [0.2,0.2]	1159	1.4 (1.3,1.4)	84999

## Discussion

Key results

This is a study that provides estimates of the risk of death and hospital admission based on the ECDS initial presentation code. This also validates previous work around the value of the NEWS2 score in predicting subsequent death. These results show that the ECDS initial presentation code has potential discriminant validity in predicting adverse outcomes of death and hospital admission. The ECDS code is well collected, as this is routinely collected as part of patient registration, we did not find much missing data. This contrasts with the final diagnosis, which is poorly recorded and limits its usefulness. There are some unexpected results, while it is unsurprising that codes indicating resuscitation (cardiac arrest) are associated with high mortality, the relatively low risk of ‘chest pain’ is unexpected. This may be a consequence of public health campaigns that encourage people with chest pain to seek medical help. This study has also identified some codes which are unexpectedly dangerous; ‘drowsy’, ‘generalised weakness’ and ‘hypothermia’, these are likely to reflect elderly patients with significant co-morbidity.

Our subgroup analysis considered initial presentations that the RCEM advocates consultant sign-off. While mortality and hospital admission are not the only reasons for consultant sign-off, our results suggest that a more data-driven approach may be worth considering. Presentations which require consultant sign-off should be identified on the basis of risk to the patient but balanced to cost. Risk does not only mean mortality, but includes other forms of harm and potential for litigation.

Our data provides supporting information about the impact of selecting presentations for senior review. Our data also confirms that abdominal pain in people over 70 years of age is a high-risk presentation with over 4% of people dying within 30 days. We could not, due to the study design, evaluate the risk of death for people who make an unplanned return within 72 hours.

Our other subgroup analysis of the association between NEWS2 score and mortality is consistent with previous work [5]. This partially validates our outcome measure. Both Masson et al. and our work show a linear increase in mortality with increasing NEWS2 scores with no particular inflection point, though we report 30-day mortality of 31% if a patient’s first NEWS2 was 9, compared to the Masson reporting 19.6%. The two studies are reporting slightly different versions of NEWS2. We reported the first recorded value, and the Masson study reported the maximum recorded value during an ED stay. We think using the first recorded score is more useful for triage decisions, though we accept anxiety and pain may increase initial NEWS2 scores and reduce predictive validity. We are uncertain whether the case mix is comparable.

Limitations

There are a number of limitations to our study. Firstly it is a single centre and it isn’t clear how our results would differ in other settings. Secondly, this is not a ‘natural experiment’, patients in this study were receiving standard medical care and the results should not be seen as describing risk for untreated patients. Our outcome variable has limitations; it is possible that a patient who lived out of our catchment area and was not followed up by our institution may have died without getting on our list of patient deaths, but any bias from this is likely to be small. However, our secondary outcome of NEWS2 scores as a predictor of 30-day mortality is consistent with previous work, suggesting that our methodology is valid. The fact that many people with a NEWS2 score of ‘No Score’ died within one day probably reflects people in cardiac arrest or peri-arrest, where clinical care took priority over recording information in the electronic health record. The outcome variables of mortality and hospital admission can be criticised as incomplete. An effective triage scale should pick up conditions at high risk of death, but also prevent morbidity. Our work does not consider conditions, such as a chemical eye injury or a dislocated ankle which have low mortality but require urgent treatment to prevent significant disability.

Implications

Our results cannot be used for automated streaming and redirection of patients who are identified as low-risk. Our patients all underwent a clinical evaluation, and this may have mitigated the reported risks. However, identifying a patient cohort that can safely be directed to alternative care would clearly be desirable. This would require validation in another cohort, ideally in a different centre and careful testing.

Triage scales are widely advocated and used in EDs but the evidence of effectiveness behind them is both limited and insufficient [3,13]. No single triage scale has demonstrated superiority over any other scale [14]. All of the commonly used triage scales are compromised by both over and under-triage of serious illness and there is clearly an 'opportunity to improve interrater reliability and triage performance' [15]. There has been some work to try and improve triage by using machine learning, but this has only been focused on suspected cardiovascular disease and we would argue that we need to consider all patients [16,17].

We anticipate as electronic health records become more established in ED care, that traditional triage models will be gradually refined and automated. Currently, there is a lack of consensus about which triage scales are good at discriminating between high and low-acuity patients [18]. While early identification of serious acute illness will always require a clinician, this is a clinical problem that could benefit from Artificial Intelligence and automation. Our data further suggests that the RCEM should consider revisions to presentations that require consultant sign-off.

## Conclusions

This study shows that the ECDS initial presentation codes are well collected with minimal missing data, this contrasts with diagnosis codes which are poorly collected. Our results also show that the ECDS initial presentation code has a good ability to predict people at high and low risk of subsequent death and hospital admission. Currently, triage tools are designed for paper use and are not automated. Our results are a useful first step in developing automated triage models that could exist as part of electronic health records. Our results show that ‘chest pain’ was not associated with a very high risk of mortality and we question whether the RCEM standard that every person over 30 years of age with a presenting complaint of chest pain should be signed off by a consultant.
